# On the Curability of Pulpless and Abscessed Teeth; Mainly by the Immediate Method with Statistics of Cases

**Published:** 1888-02

**Authors:** George Cunningham


AMERICAN JOURNAL

—OF—

DENTAL SCIENCE

VOL. XXI. THIRD SERIES—FEBRUARY, 1888. No. 10.

ARTICLE I.

### ON THE CURABILITY OF PULPLESS AND ABSCESSED TEETH; MAINLY BY THE IMMEDIATE METHOD, WITH STATISTICS OF CASES.

BY GEORGE CUNNINGHAM, B. A., D. M. D., L. D. S. ENG.

[Being a Paper read before the Dental Section of the International Medical Congress, Washington, 1887.]

The principal work which I wish to lay before this Section, and that to which I attach the most importance, are the statistics. In presenting such a record of a large number of cases, it is possible for everyone to examine them in detail. I shall now hand round among the members copies of the actual records from which the tables are prepared, so that they may have an opportunity of examining them, and may be convinced that the figures, carefully compiled from these records, have a reliable foundation and basis. The work has proved of such a Herculean nature that I think I shall be excused for saying that I have not exhausted all the information derivable from the tabulated records, and that the present communication might be taken as an earnest of my sincerity and interest in the investigations. The relativity of the various teeth to the condition of being pulpless or abscessed is shown by these tables, but time will not permit of my entering on that subject now. The system on which the records were kept was embraced in a paper * I read before the American Dental Society of Europe, and has been employed for several years with success by myself and others. I should like to observe that when these cases were recorded I had not the slightest intention whatever of preparing a paper on this subject. The large detailed tabulations are simply the exact copies of the everyday record of *each* and *every* case as it came under notice. From the attention which the Immediate Method has recently received in the *Cosmos* and other American dental journals, and from the disbelief with which Dr. Craven's paper has been received, I am more glad than ever that I felt impelled to tabulate the results of this method and place it before this Section of the International Medical Congress.

The troublesomeness, the tediousness, and the disasters attending the treatment of pulpless and abscessed teeth are only too well known. The literature on the subject was so very extensive, and the views expressed so very divergent, that a perusal of it was absolutely perplexing. Peculiarly interesting and severe cases had been reported in detail; but no writer on the subject had yet given any general statistics illustrative of the results of ordinary practice. It would be only fair, however, to mention one exception, more especially as it was from the conviction carried by the clinical notes of these cases that I was induced to adopt the present line of treatment. It mainly arouse out of a criticism of his book on "Dental Surgery" that Mr. A. Coleman prepared a paper which was read

* "A Suggested System of Dental Notation for the Use of Dentists in Recording Operations." Dental Manufacturing Company, London.

before a meeting of the British Dental Association, on October 14th, 1882. It was a paper on the treatment of "dead teeth" (as he called them) by an antiseptic process. He says :—

"Such cases may be briefly described as teeth in which the whole, or nearly the whole, of the dental pulp has lost its vitality, and where the adjacent dentine, either through decomposition of the contents of the dentinal tubuli, or saturation with the septic fluids from the decomposed pulp, has become putrid and offensive, and, according to the degree of its putrescence, coupled with the state of health of the individual, more or less affects the cementum and its contiguous vascular membrane."

Mr. Coleman, in describing the process which he recommended for the generality of such cases, says :—

"After removing all the softened dentine and the contents of the pulp cavity, but not those of the fangs, and well syringing and drying, carbolic acid on cotton is placed in the pulp cavity, and left there for a few minutes, the time being generally occupied whilst preparing the filling. This latter being made ready, the carbolic acid is then removed, and the cavity again dried, and over the fine cavity or cavities, as the case may be, is placed a small disc of stout writing paper moistened with carbolic acid, on one side of which has been taken up the twentieth to the fifteenth of a grain of arsenic, this side being applied to the fang cavity. Over this zinc-oxychloride, as usually mixed for a filling, is placed as much as nearly or quite fills up the pulp cavity; and when this has set, the remainder of the cavity may be filled with any suitable filling. In the case of molars, and where it may be supposed there is any possibility of a second application of arsenic being necesssry, I fill temporarily with gutta percha."

Mr. Coleman then proceeded to give the records of a certain number of cases; and it was from those cases being reported so fully that I had the confidence to adopt that operation. I must explain that my practice is in a university town, and I have therefore a very peculiar practice—a constant succession of patients who come and then disappear. It must not be assumed that these patients necessarily went away because of the ill effects of my treatment, but because they removed to other spheres on the conclusion of their academic career. I purpose making an effort to complete the history of those cases, and to get a much fuller report as to the cases which have not been re-seen. As these proceedings will be reported in the English journals, I appeal to the profession to assist me, when possible, in filling out the history of any case I have reported, especially by sending notice of my failures. The intractable nature of many patients had led me to endeavour to get over the difficulty, especially with these troublesome students, by treatment at the one sitting; because otherwise, before the dressings had come to an end, the patient would have disappeared. I could not make up my mind to continue what I considered at best a somewhat filthy kind of practice. I was too strongly imbued with the training I had received in this country not to appreciate the fact that, contrary to what Mr. Coleman advised before I did anything I should remove the contents of the pulp canals more or less thoroughly, and that any extra expenditure of time which this operation demanded would be well repaid. The consequence was that I endeavoured to make a compromise between the two plans.

I may say that in many instances in my practice the pulp canals have never been completely filled. A certain percentage of our fellow-practitioners, as was known from their own statement, did things perfectly; but a great number did not do things perfectly. I belong to the latter category. By referring to the diagram which I have drawn upon the black board it may be seen that when I do not know to what extent the root or roots of the tooth were filled, I try to form some conception of it by means of a similar diagram in the case book. It is only in very rare cases, where the root canal is unusually patent, that I have found it necessary to extend the cleansing process to the immediate neighbourhood of the apex of the root. All root canals which were found too samll to admit a fine nerve bristle were left untouched. When we remember the frequency of curved and other irregular formations of the ends of the roots, it might be well to recognize the fact that the operative difficulties of complete removal to the very apex must often be insurmountable, and that from the danger of perforating the root at some other spot than the apex there may be a danger in over-thoroughness of excavation.

When I was on the Continent last year visiting the German dental schools, I had the opportuuity of inspecting the interesting school of Leipsic under the care of Professor Hesse. We very quickly found out that each was anxious to communicate something of importance to the other, and that was as to the Immediate Treatment of teeth. Professor Hesse was carrying out that system in his school, and was doing so with success.

The present record of cases, treated by the immediate method, went back as far as the earliest part of 1883, soon after the publication of Mr. Coleman's paper. Before discussing the record, I would call your attention to one case in particular. I was going to operate for a personal friend, and having put on the rubber dam, I had just removed a zinc phosphate filling from the superior right second bicuspid, thereby exposing the putrescent pulp, and was about to clear out the carious cavity, when a very urgent message came to me from another patient. The gentleman on whom I was operating said he would take it as a favour if I would attend to the message and let him go. I did so, after removing the rubber dam, without even inserting a dressing, so you can imagine my surprise when he appeared next day with as big a face as any I have ever seen in the whole course of my professional experience. If a filling or even a dressing had been inserted, I would have ascribed this condition to the occluding and shutting up of the putrid matter. As it could not possibly be due to that, this case seems to indicate that the sudden development of a so-called blind abscess into an acute one is not necessarily due to the occlusion of the root canal, nor to the passage of putrid *debris* beyond the apex of the root.

As my friend was an eager experimentalist, he encouraged me to carry the test of the immediate method further than I had hitherto ever dared. On Nov. 8th, 1884, the second day after the removal of the stopping, while the abscess was in this acute stage and the face much swollen, I applied the rubber dam, cleansed the cavity and root canals, inserted a minute dressing of arsenious acid and oil of cloves, and filled each root-canal with zinc oxychloride, finishing the main cavity with Ash's phosphate cement. After removing the rubber dam, the gum was lanced. The case progressed favorably, and the tooth has never given any subsequent trouble.

The various agents employed under the Dressing Method, were eucalyptus oil, the same combined with iodoform, creosote and iodoform, oil of cloves, carbolised resin, tincture of aconite, and oxychloride of zinc. I have a record of 122 teeth so treated—the majority of them (seventy four) having been treated with eucalyptus oil or with eucalyptus and iodoform. The number of extractions which I was obliged to make in those cases is six— a percentage of 4.918. I do not claim that as the total percentage of teeth extracted under the Dressing Method, because I am sure that there is a certain number of cases the further history of which has been lost, and which, no doubt, includes several extractions. But at the same time that figure, small as it is, will be sufficient for the purposes of comparison with the results of the other method. The next thing which has been noted is the number of subsequent occasions when the patient had returned, generally in the course of having other teeth treated, complaining of slight inflammation of the periosteum. This number is thirty-six, or a percentage of 29.5; while the number of subsequent swollen faces and abscesses is thirty-two, or a percentage of 26.2. The next point of importance noted is the number of permanent stoppings inserted at the time of filling the root canals. It was found that in the years 1883-4 there were only two such out of thirty-eight cases, or a percentage of 5.26.

In the course of the Immediate Method, the first dressings employed were arsenious acid and oil of cloves. The modification of Mr. Coleman's plan, which I adopted, was simply to take the merest shred of cotton wool on a fine nerve bristle, the difficulty being to get this shred small enough. I am ready to admit that even this amount of cotton wool was a disadvantage, but I had not found anything which would do better as a vehicle. Having dipped the wool in oil of cloves, I simply touched the end with the smallest possible portion of arsenious acid, and carried it up as far as I thought it safe to go in the direction of the apex of the root. Finding that that was occasionally an unsatisfactory method in other hands, I feel that I ought to give a warning and a caution about it. I have the incomplete and careless records of one assistant, who acted as *locum tenens* for a time—an otherwise able man, who contributed to the literature of the profession, and who thought he knew all about this treatment at once. If I were to collate the extractions and the extensive necroses which followed that assistant's treatment, the members would certainly condemn this use of arsenious acid; but, in the proportions which Mr. Coleman had advised as quite permissible in the pulp cavity (a few milligrammes), the amount was so small that no deleterious results followed. It was evident that too large a proportion of arsenious acid had been used, and in several cases I found that the root had been peforated and the dressing forced beyond the opening. The results which followed were only what might have been expected from such careless and reckless manipulation. It was impossible to make out a record of the work of the *locum tenens* above referred to, as he had failed to inscribe the necessary data, evidently thinking such a tax on his time was superfluous. In none of the cases treated by my brother or myself has there been, so far as I know, a single case of loss of a tooth due to an overdose of the arsenious acid.

Not from any failure by the employment of the first method, but in an endeavour to adopt a more exact method I next employed a solution of arsenious acid in glycerine. By careful and prolonged manipulation over a sand or water bath, I made a 1 per cent. solution, which answered, admirably, and has been very favourably commented on by several other practitioners who have used it. My brother got a chemist to make up for him a solution of arsenious acid in alcohol, with oil of cloves, which formed a 2 per cent. solution.* After a time it was desired to test the efficacy of bichloride of mercury in this method, and, therefore, a solution of one in 1,000 had been employed in forty-five cases, and another of one in 100 in seventy-five cases. The 1 per cent. solution of arsenious acid had been employed in 165 cases. Eucalyptus oil, I am confident, would also prove efficient; while eucalyptus oil in combination with iodoform and oil of cloves had been used in a few cases. I have also tried dressings of a mouth wash, consisting of thymol, benzoic acid and tincture of eucalyptus in water, which was published as emanating from Professor Miller, of Berlin, in one or two cases.

In order to prevent possible erroneous conclusions from these facts, it may be well to state that the decision to employ the mercuric chloride in place of the arsenious acid was not arrived at from any dissatisfaction with the latter, but from a recognition of the enormously greater

---

| | |
|---|---|
| Acid, Arseniosi - - . - | gr. ii. |
| Sp. Vini Rect - - - - - | ʒi. |
| Ol. Caryoph. - - - - - | ʒi. |

Misce.

If the arsenious acid does not dissolve at once, it will do so in time Gentle heat over a sand bath will promote solution.

power of the former over the latter as an antiseptic and germicide. Indeed, whilst I was trying the mercuric chloride, the use of arsenious acid was continued both by my brother and an assistant.

The mode of use of the mercuric chloride is very simple. After the preparation of the root canals, they are well syringed out several times with the 1 per cent. solution. During the preparation of the instruments, the zinc oxychloride and the cotton wool dressings for carrying the latter well into the root, the canals are left soaking in the solution. The excess only is then absorbed, thus leaving the walls of the canals moist, a matter of some importance, as if they are wet the zinc oxychloride will penetrate further into the finest canals than if they are dry. The addition of mercuric chloride to the first oxychloride mixed in this way is no disadvantage, but is possibly unnecessary.

In the absence of any generally recognized scientific classification of pulpless and abscessed teeth, these cases have been tabulated under three distinct conditions, viz.:

(*a*). Those where the pulp was removed at the time in a fairly healthy or non-putrescent condition.

(*b*). Those where the pulp, or what remained of it, was in a putrid state, including, therefore, all cases of so-called blind abscess.

(*c*). Those where a fistulous opening indicated with certainty the presence of an apical abscess.

Out of a total number of 512 teeth treated by the Immediate Method, the total number of known extractions was three. Two of these had been marked at the time before beginning the operation as "forlorn hope;" and one of them was removed partly for artificial work. The tooth was loose. My assistant had made a very pardonable mistake. It was a left superior bicuspid with that rather uncommon abnormality a bayonet-shaped root. After its extraction it was found that in drilling out the root canal the instrument had perforated the root just at the bend, and the arsenical clove-dressing was found protruding through this perforation, thus setting up a chronic periodontitis, whether by acting as a mechanical or a chemical irritant, but not improbably as both, is uncertain. One tooth which had been extracted has not been included in this list, for the following obvious reason. On 19th November, 1884, my brother treated and filled with the arsenious acid and oil of cloves and phosphate cement a right upper lateral incisor for a lady student at Newnham College, and on 5th February, 1885, he filled the tooth permanently with gold. On the 25th June, 1886, whether by overstudy, by excess of exercise, or from what cause we know not, the hitherto absent right upper canine tooth was erupted directly over the lateral incisor. It therefore became necessary to sacrifice one tooth or the other; and it was deemed better to extract the lateral incisor. Under these circumstances it would be an error to include that extraction as coming among these cases.

The next point to which I would call your attention is the number of subsequent cases of slight periostitis calling for treatment under this immediate method. My record shows six cases as against thirty-six under the dressing method, or a percentage of 1.152, as against a percentage of 29.5. Under the Immediate Method the number of cases of swellings and abscesses treated subsequently was five, or a percentage of .976 as against thirty-two, or a percentage of 26.2. I do not ask members to take these percentages as absolutely exact; but as they were calculated from tables in which both methods had been recorded with equal faithfulness, so far as statistics went, they represented faithfully the relative advantages of these two distinct methods of treatment. There was no partiality or favour shown to the one system more than to the other. Under the dressing system in 1883-4, only two teeth out of thirty-eight, or a percentage of 5.26, had been permanently stopped at the time of filling the root canals, whereas under the immediate method in 1886-7 sixty-one teeth out of 150, or a percentage of 40.66, had been permanently filled at once. That surely showed in the most emphatic manner the gradual conviction to which I have come to as to the results obtained by the Immediate Method in my practice. The rubber dam was recorded as having been used in 200 out of 270 cases, or in about 75 per cent. for the Immediate Treatment, and in 52 cases out of 153, or about 33 per cent. for the dressing method. In order to give some idea as to the number of cases which I have seen again, my records are not fully made up; but in one case book, out of a total of 114 cases, 70 of them had been seen again; in another, out of 109 cases, 66; and in a third, out of 49 cases, 40. I believe, therefore, that the extractions under the Immediate Method (taking into consideration the number of cases seen again, and making a liberal allowance for the patients who have consulted some other practitioner) have not exceeded 2 per cent. Making a still larger allowance for the patients in pain who have sought other advice in cases of subsequent acute periodontitis, I should say that the number was about 3 per cent., but possibly less.

The success of the operations seemed mainly to depend on the old axiom *sublata causa tollitur effectus.* No observant practitioner can have failed to notice the inherent curability of numbers of abscessed teeth. Who has not noticed the frequency of cicatricial tissue marking the existence of former fistulous tracks, even where the putrefactive contents had been allowed to remain? It was to this inherent property of spontaneous curability that we look for the relief and cure of all the injurious conditions arising from a putrescently diseased pulp. In my opinion the best mode of sterilization for infected dentine is excavation; therefore I use the nerve drill freely where the presence of micro organisms is suspected. By removing the greater part of the diseased tissue, even although the naked eye might not be able to detect the existence of any diseased process, accompanied by micro-organisms, they might fairly anticipate that the strife between diseased processes and healthy action would come to an end. If the general condition of the individual were good, such a case would probably have a favourable termination. Is it not possible that the pathogenic functions of micro-organisms in these conditions are exaggerated? It should be remembered that many of these organisms found in the mouth and tooth cavities were non-pathogenic. Certainly, a large number, if not the majority of recorded cases, could be treated by the removal of all fairly accessible degenerated pulp tissue and infected dentine; and that, with the complete occlusion of the cavity, would result in a cure (unless there was a distant discharge of pus from the apex, or unless a fistulous track was present.)

The impossibility of diagnosing with any absolute certainty the existence of an abscess sac at the apex of the root had to be recognised. On the occlusion of the pulp canal or canals of such a tooth, a slight amount of subsequent inflammation should be regarded rather as an aid than as an obstacle in the complete removal of the existing conditions, while a more acute inflammation would ultimately promote the complete absorption and removal of any old existing inflammatory product. In a large number of cases it is probable that merely a quiescent condition is established. The pain and constitutional disturbances arising in the more severe cases of inflammation might be successfully combated by the employment of local counter irritants, and depletion, aided by aperients and the internal administration of some such drug as quinine, which possessed the power of diminishing the activity of the inflammatory process—whether by checking the activity of the cells, by diminishing the exudation, or by reason of the oxidation process, I do not know. But I can vouch for its extreme use and benefit in those conditions; and I can also vouch for the subsequent gratitude of patients (when they got into a state of despair) to the dentist for such remedy, instead of meekly yielding to the not unnatural desire for extraction.

In the majority of cases the inflammatory process was so slight as to pass unnoticed, or was not excited at all; and yet the existence of an abscess sac might be assumed as being present in many of them. In most cases it might be anticipated that either the walls of the sac adhered and became united together, or that the contents gradually dried up and became converted into a caseous mass, in which state it was absolutely innocuous and perfectly harmless, remaining where it was.

In the very small number of cases where the diseased condition persisted, resort might be had to the heroic treatment by the burring of the necrosed part of the root and alveolus, or by the injection of aromatic sulphuric acid into the diseased part. In several cases, where extensive excavations of the alveolus had occurred, it might be assumed, from their healing so rapidly, that granulation tissue had formed in the interior of the sac, followed by a gradual contraction of the newly formed tissue, converting it into a small knot of old cicatrized fibrous tissue.

I believe that the operation called rhizodontropy and the insertion of a drainage tube within the tooth were unnecessary. Rhizodontropy might afford a temporary and convenient relief to the patient, and (should I say it?) to the busy operator; but in several cases I have seen it result in a renewal of the abscess, or in the loss of the tooth.

Finally, no matter how much these suggestions as to the pathological conditions of pulpless and abscessed teeth and the changes produced by treatment might be subject of debate, there could be no question as to the silent eloquence of the facts showing the results of treatment as set forth in the statistics of cases which I have presented. Contrasting, then, the relative advantages of the dressing method as compared with the Immediate Method of treatment, I am led to the following conclusions:—

1st—That under the Immediate Method there were fewer extractions and failures.

2nd—That there were fewer subsequent attacks accompanied by swellings and acute abscess, and therefore the Immediate Treatment was attended with less pain.

3rd—That it required a considerably less expenditure of time on the part of both the patient and operator, the average time of treating and filling such teeth being considerably under an hour.

4th—That in consequence of these considerations, we were able to treat and were able to save more desperate cases, many of the cases mentioned in the record having large perforations of the roots, while others had been already condemned by other practitioners as utterly hopeless.

5th—That method, rather than medicine, had a good deal to do with the results, and that probably the operation would have succeeded equally well in a very large number of cases without any medicine whatever.

6th—That, from the difficulty of diagnosing such cases, it is better to conduct every operation with all antiseptic precautions.

7th—That casualties, such as perforation of the root, under the Immediate Method of Treatment, had been fewer, probably because of the less complete removal of the contents of the root canals.

Conscious, as I am, of the incompleteness of the record in some respects, I hope at some future time to complete the work, and meanwhile present a few actual tracings from my case book, some of them showing desperate cases where I scarcely anticipated success, and which I could only treat, either in justice to my patient or with satisfaction to myself, by the Immediate Method.—*The Dental Record.*

